# An Interdisciplinary Approach: Presentation of the Pediatric Obstructive Sleep Apnea Diagnostic Examination Form (POSADEF)

**DOI:** 10.3390/diagnostics14151593

**Published:** 2024-07-24

**Authors:** Janine Sambale, Richard Birk, Ulrich Koehler, Wulf Hildebrandt, Heike Maria Korbmacher-Steiner

**Affiliations:** 1 Department of Orthodontics, Clinic of Dentistry, Philipps-University Marburg, Georg-Voigt-Str. 3, 35039 Marburg, Germany; 2 Department of Otolaryngology, Philipps-University Marburg, 35043 Marburg, Germany; 3 Department of Pneumology, Philipps-University Marburg, 35043 Marburg, Germany; 4 Department of Anatomy, Philipps-University Marburg, 35037 Marburg, Germany

**Keywords:** pediatric obstructive sleep apnea, craniofacial anomaly, dysfunction, examination form, interdisciplinary diagnostic

## Abstract

This report emphasizes the need for interdisciplinary collaboration in diagnosing and treating pediatric obstructive sleep apnea (OSA). OSA, affecting 1% to 4% of children, often results from adenotonsillar hypertrophy, craniofacial disorders, or obesity. While adenotonsillectomy is the primary treatment, about 75% of children, especially those with craniofacial disorders or obesity, continue to experience OSA symptoms post-surgery. To address these cases, several medical fields emphasize the necessity and demand for interdisciplinary collaboration in managing pediatric OSA. Therefore, the authors aimed to develop the Pediatric Obstructive Sleep Apnea Diagnostic Examination Form (POSADEF). This form, based on clinical experience and the literature, captures craniofacial and functional characteristics linked to pediatric OSA. A case study of an eight-year-old girl with OSA, who was unsuccessfully treated with adenotonsillectomy, underlines the importance of the diagnostic examination form. The orthodontic assessment revealed craniofacial disorders and subsequent treatment with maxillary expansion and functional appliance therapy resolved her OSA symptoms. This case demonstrates the value of POSADEF in enabling comprehensive evaluation and treatment across medical disciplines. POSADEF is designed to assist health care professionals in diagnosing craniofacial and orofacial anomalies contributing to pediatric OSA.

## 1. Introduction

Obstructive sleep apnea (OSA) in children (2–18 years) is a sleep-related breathing disorder (SRBD) characterized by intermittent partial or complete upper airway obstruction [1]. The prevalence of pediatric OSA is estimated to range from 1% to 4%, with adenotonsillar hypertrophy being the most common causative risk factor [2]. Additionally, there is an increased prevalence of OSA among children with craniofacial disorders and obesity, with obesity itself increasing in incidence during the last few decades [3]. Adenotonsillectomy remains the first-line therapy in pediatric OSA, with approximately 70% of children showing good results [4]. However, OSA persists after adenotonsillectomy in almost three-quarters of children with craniofacial disorders or obesity [4,5,6]. The consequences of untreated OSA are significant, including impaired cognitive function, academic performance, increased risk of depression, hypertension, diabetes, and a 226% increase in health care utilization [7,8,9]. The persistence of OSA after surgery is attributed to the fact that it does not address underlying causes such as low muscle tone or craniofacial disorders [6]. An interdisciplinary approach involving pediatricians, otolaryngologists, and orthodontists in the diagnosis and treatment of pediatric OSA has been demanded [10].

Therefore, we aimed to present our novel Pediatric Obstructive Sleep Apnea Diagnostic Examination Form (POSADEF), which is designed to provide interdisciplinary standardization in the clinical examination of children with OSA. The successful multidisciplinary treatment of a young girl with OSA and underlying craniofacial anomalies and orofacial dysfunctions illustrates the significance of interdisciplinary collaboration.

## 2. Development of the Pediatric Obstructive Sleep Apnea Diagnostic Examination Form

Due to the multifactorial nature of pediatric OSA, coupled with the high prevalence of persistent OSA after adenotonsillectomy, the need for increased interdisciplinary collaboration has been raised by the medical departments to effectively treat children with OSA [6].

**Figure 1:**
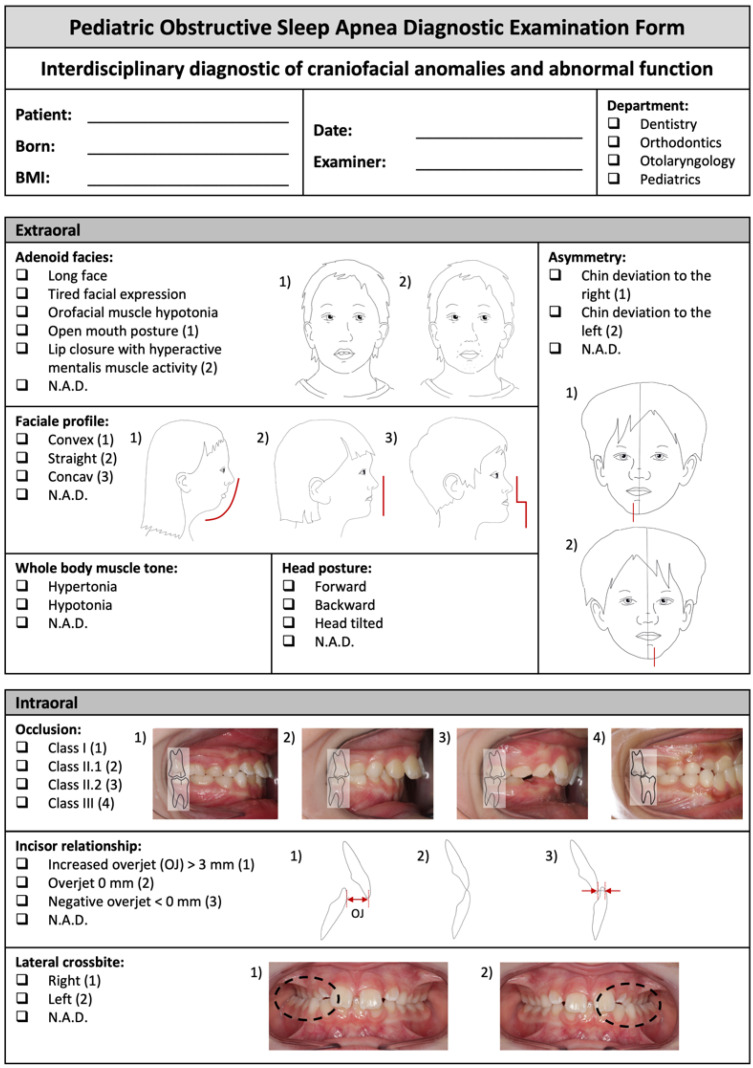

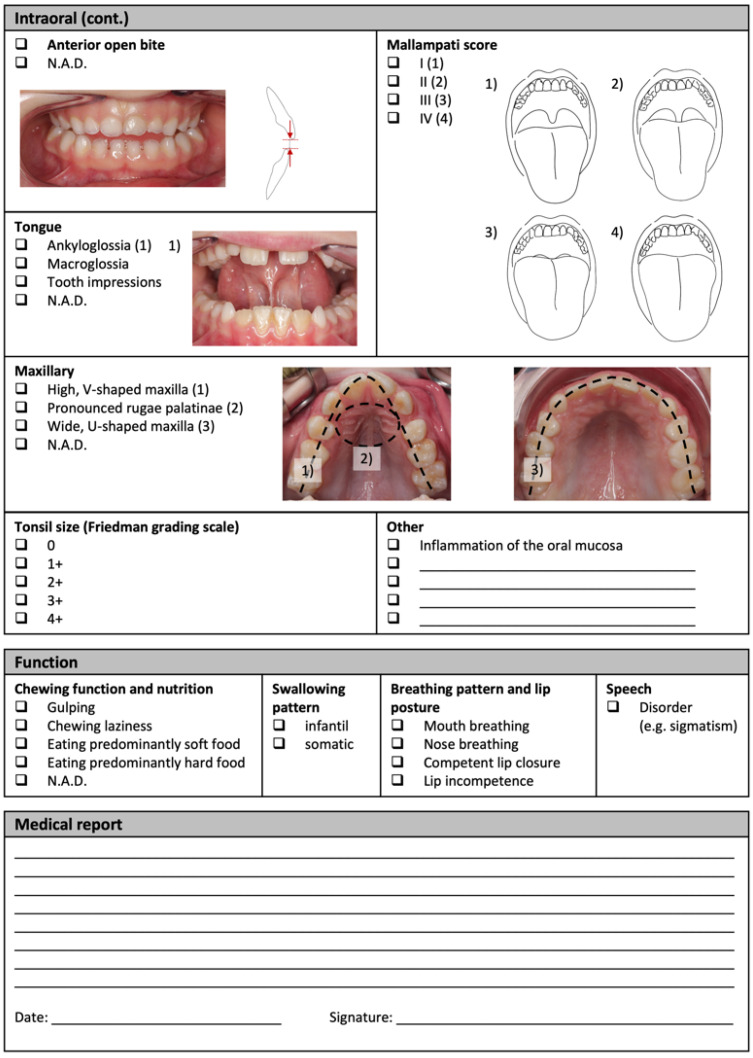
Pediatric Obstructive Sleep Apnea Diagnostic Examination Form (POSADEF), pages 1 and 2. Abbr.: N.A.D. = no abnormality detected, BMI =body mass index.

Based on clinical expertise and a literature review (Table 1), the authors developed an examination form (Figure 1) to enhance communication among dentists, orthodontists, pediatricians, and otolaryngologists involved in diagnosing and treating pediatric OSA. This examination form integrates dental, orthodontic, myofunctional, sleep medical and otolaryngological aspects, reflecting our interdisciplinary approach. The interdisciplinary team consists of trained specialists in dental sleep medicine, sleep medicine, and an otolaryngologist. Constructed from both dental and medical perspectives, the examination form incorporates aspects of speech therapy, considering the close collaboration between dental sleep medicine specialists and speech therapists. Drawing from clinical experience and the literature, we structured the examination form’s sections to capture craniofacial characteristics and functional features associated with pediatric OSA.

The risk factors and associated features are included in the examination form (Figure 1).

The layout follows a clear, logical structure incorporating an interdisciplinary, easy- to-understand terminology. The questionnaire consists of two pages and is used to record nominal, metric and the relevant craniofacial, orofacial and functional data (Figure 1). Page 1 (Figure 1) outlines extra- and intraoral findings. To enhance comprehensibility, we replaced Latin terminology with more accessible terms where necessary. A proclined or retroclined head posture is, for example, described as “forward” and “backward”.

The extra- and intraoral orthodontic aspects were illustrated and highlighted with additional drawings (e.g., occlusion, incisor relationship, lateral crossbite).

Page 2 (Figure 1) integrates intraoral findings with orthodontic and otolaryngological parameters (e.g., Mallampati score and tonsil size). The different palatal arch sizes are represented in two figures, with the Latin terminology “rugae palatinae” retained and clearly marked. To better understand this, the authors replaced the original terms “high narrow palatal arch” and “flat wide palatal maxilla” with “high, V-shaped maxilla” and “wide, U-shaped maxilla”. Functional aspects (e.g., chewing function, swallowing, and breathing patterns) are also integrated on page 2. To illustrate the significance of knowledge regarding craniofacial and orofacial anomalies, as well as functional aspects associated with pediatric obstructive sleep apnea, the authors present a case report of a young patient who did not benefit from adenotonsillectomy as the sole treatment approach.

## 3. Case Report

A young girl with pediatric obstructive sleep apnea underwent tonsillectomy by an ENT surgeon at the age of six years. One year later, due to persistent sleep-related breathing symptoms and recurrent otitis media with hearing deficits, the outpatient pediatrician referred her to the Department of Otolaryngology at the University of Marburg for further evaluation. Besides persistent OSA, the otolaryngologist diagnosed an Eustachian tube dysfunction (ETD) and treated her with an Eustachian tube balloon dilatation. After diagnosing a high and narrow palatal arch, the otolaryngologist referred her at eight years of age to the Department of Orthodontics for further evaluation.

The young patient still showed symptoms of sleep apnea, with her mother reporting persistent snoring, abnormally loud breathing with episodes of apnea, limited nasal breathing, and sleepiness during the daytime. The sleep-related breathing disorder scale of the pediatric sleep questionnaire (SRBD-PSQ) revealed a value of 0.59 (cutoff value > 0.33) [23].

**Figure 2:**
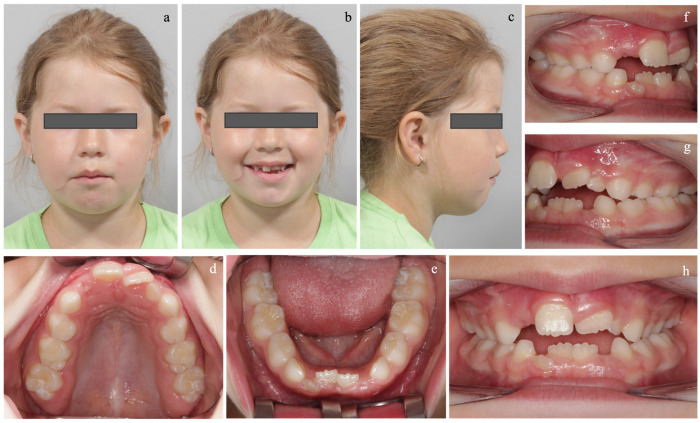
Pretreatment extraoral examination: (**a**) Lip incompetence with orofacial muscle hypotonia, hyperactive mentalis muscle activity, fatigued facial expression, and deviation of the chin to the left. (**b**) Excessive buccal corridor anticipating maxillary constriction. (**c**) Excessive convex facial profile. Pretreatment intraoral examination: (**d**) High narrow palatal arch with pronounced rugae palatinae. (**e**) Low tongue posture. (**f**,**g**) Class II.1 malocclusion. (**h)** increased overjet (9 mm) and a lateral crossbite to the left.

The extraoral and intraoral examinations are presented in Figure 2. The functional examination revealed a backward head and open mouth posture with orofacial muscle hypotonia and mouth breathing. Furthermore, she exhibited an interdental and addental stigmatism with a tendency for an open bite.

**Figure 3:**
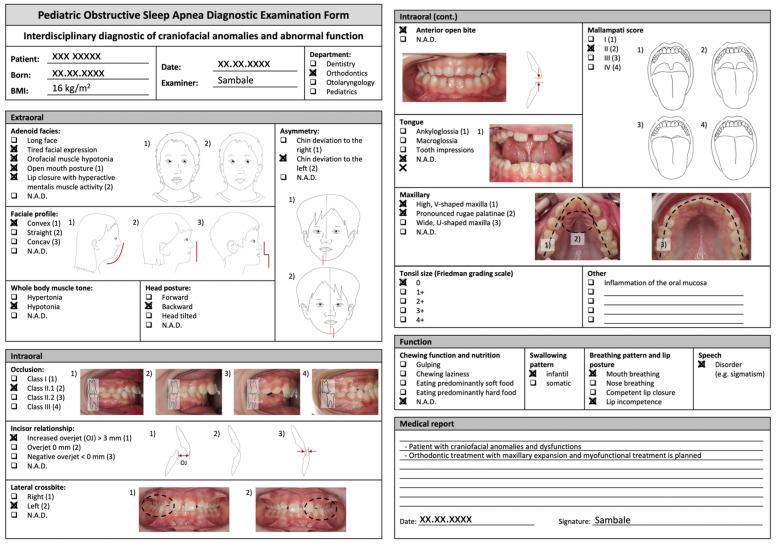
The filled-in POSADEF showing the patient’s diagnosed craniofacial characteristics and features.

The POSADEF was filled in according to the characteristics and features mentioned above (Figure 3).

**Figure 4:**
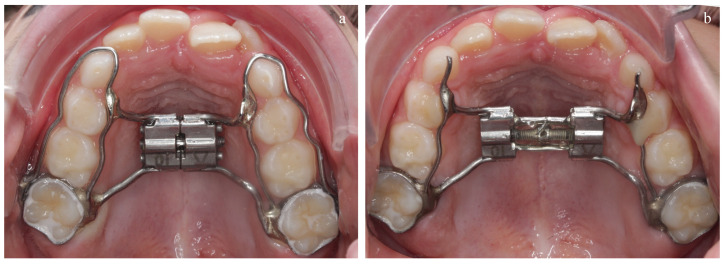
Maxillary expansion with a Hyrax expander. (**a**) Before transversal expansion. The lateral primary teeth were lingually and buccally strengthened by a curved wire. (**b**) After transversal expansion. The curved wire loop was cut buccally and palatally fixed with a composite at the primary canines.

The initial treatment approach involved maxillary expansion with a Hyrax expander to widen the narrow palatal arch and correct the unilateral crossbite. The Hyrax expander was used with a jackscrew (length: 10 mm; Dentaurum) and two bands on the first molars (Figure 4a). The expander screw was activated at a rate of a one-quarter turn (=0.20 mm) per day after the appliance had been inserted. Activation after 37 days was discontinued when the lingual cusps of the upper first permanent and deciduous molars contacted the buccal cusps of the lower first permanent and deciduous molars (Figure 4b). After that, the mandible was sagittally unlocked and showed an improvement in the molar relationship with an advancement of two millimeters. Additionally, the SRBD-PSQ showed a remarkable treatment response with a value of 0.14. The patient showed significantly improved nasal breathing, no snoring or apnea sounds, and was no longer sleepy during the day.

**Figure 5:**
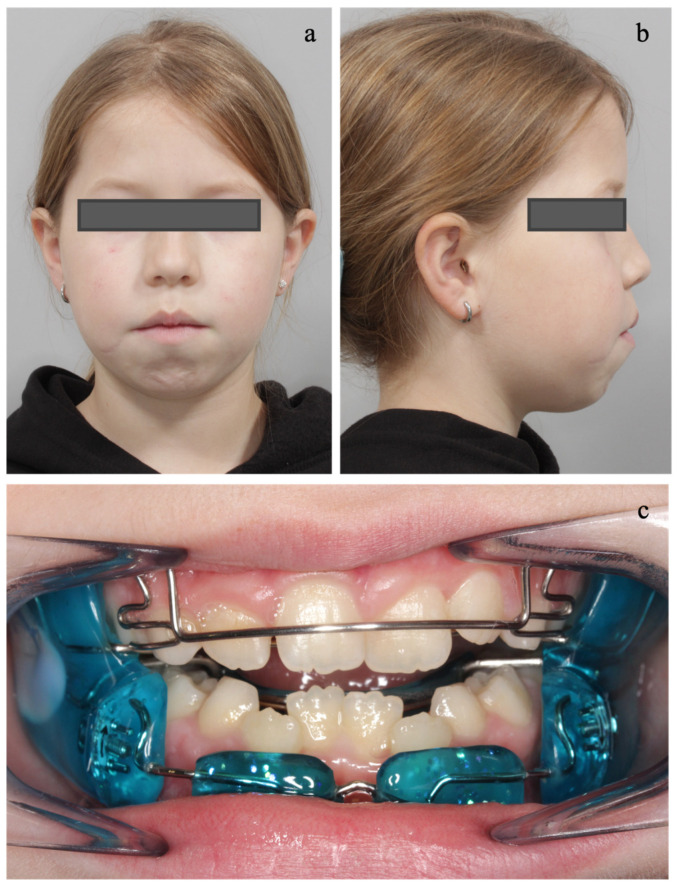
Extraoral and intraoral photographs with the Fränkel 2 (FR-2). (**a**) Enface with the FR-2 in situ showing hyperactive mentalis muscle activity. (**b**) Profile with the FR-2 in situ showing hyperactivity of the orofacial muscles with the aim to strengthen the hypotonic orofacial muscles. (**c**) Intraoral photograph with the FR-2 in situ.

After treating the skeletal transversal discrepancy and after six months of retention, the patient was referred to a speech therapist in order to correct their tongue posture and sigmatism. Simultaneously, functional appliance therapy was conducted with the Fränkel 2 (FR-2) appliance. The vestibular buccal shields and the lower labial pads restrained the musculature and removed the restricting muscular forces from the upper and lower jaws. In the maxillary region, the buccal shields were constructed to stabilize the maxilla after transversal expansion, and the labial pads enhanced physiological mandibular growth, restraining the mentalis muscle. The aim was to strengthen the hypotonic orofacial muscles (Figure 5a,b). The therapeutically desired jaw relation was three-dimensionally registered with a wax construction bite (Figure 5c). The patient was motivated to wear the appliance for more than 12 h/day. During the daytime, the FR-2 appliance served as a training device to establish lip competence and improve muscle tone.

**Figure 6:**
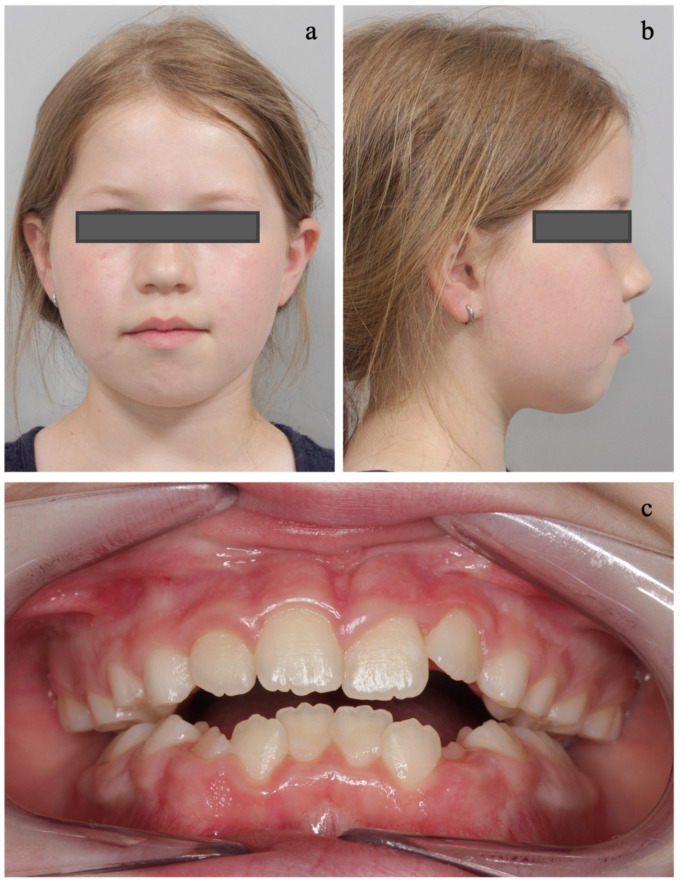
Post-treatment extraoral and intraoral examination. (**a**) The extraoral examination revealed lip competence with nose breathing and harmonization of the orofacial muscle tone. (**b**) The convex facial profile was improved. (**c**) Intraoral examination showed the stability of the maxillary width with the lingual cusps of the upper first permanent and deciduous molars contacting the buccal cusp of the lower first permanent and deciduous molars.

The extraoral and intraoral results after FR-2 treatment are presented in Figure 6. The facial profile benefited both from maxillary expansion with jumping-the-bite of the mandible and from the FR-2 with functional harmonization of the orofacial muscle tone (Figure 6b). Tongue posture was improved, and the maxillary width remained stable (Figure 6c). Since this treatment was an early orthodontic intervention, it aimed to correct the skeletal jaw relationships accompanied by myofunctional therapy to ensure the physiological growth of the jaw. Subsequent orthodontic treatment may become necessary, which is why the patient was included in a six-monthly recall to closely monitor further craniofacial growth and the stability of myofunctional harmonization.

## 4. Discussion

This report responds to the demands of interdisciplinary collaboration in children with OSA. The presented case report of a young patient with persistent OSA following adenotonsillectomy is one among many cases of children with craniofacial or orofacial anomalies who were referred to our orthodontic department with persistent OSA symptoms after adenotonsillectomy. This case is consistent with the literature, demonstrating that the presence of phenotypes (shown in Table 1) other than adenotonsillar hypertrophy highlights the necessity of interdisciplinary collaboration as a key factor in the management of pediatric OSA [6].

The diagnostic accuracy of pediatric screening questionnaires has already been evaluated and compared to polysomnography as the reference standard for the diagnosis of OSA in children [24,25]. A recent systematic review showed that the sleep-related breathing disorder scale of the pediatric sleep questionnaire (SRBD-PSQ) is a sensitive tool for detecting pediatric OSA [24]. However, a limitation of these questionnaires is their symptom-based screening. For an interdisciplinary treatment approach to pediatric OSA, an additional clinical examination might be essential to address its underlying etiological causes.

With the introduction of this newly developed examination form (Figure 1), the authors aim to promote its clinical utility and contribute to the establishment of standardized interdisciplinary diagnostics to accurately identify the pediatric OSA phenotype (Table 1).

Adenotonsillar hypertrophy is commonly thought to be the predominant phenotype of airway obstruction in pediatric OSA [2]. The Friedman tonsil grading system [26] (Figure 1) may, therefore, be a useful tool for both dentists and physicians to evaluate the size of tonsils. Because tonsil size does not correlate with OSA severity, there is no absolute cutoff point for tonsillar hypertrophy to necessitate a referral to an otolaryngologist for further evaluation [27].

However, it is important to note that adenotonsillar hypertrophy is a physiological response in childhood and may not necessarily be associated with pediatric OSA. As the skeletal boundaries of the airway expand, the lymphatic tissues in the upper airway (e.g., tonsils and adenoids) undergo regression. The combination of skeletal growth and soft tissue reduction leads to substantial enlargement of the upper airway throughout infancy, childhood, and adolescence. These alterations in airway dimensions resulting from natural growth surpass any influence of orthodontic or orthopedic interventions on airway morphology. Understanding these developmental shifts is crucial to comprehending the dynamics of pediatric OSA because respiratory disorders in children often involve a multifactorial occurrence [6,28].

Another phenotype and risk factor is obesity, which is showing increased prevalence in children [3]. Increased fat in the pharyngeal soft tissue reduces the pharyngeal airway space and increases collapsibility. Consequently, obese children face an increased risk of developing OSA compared to their normal-weight counterparts [29].

Other OSA phenotypes, which are presented in our examination form (Figure 1), include craniofacial and orofacial anomalies as well as dysfunctions and impaired muscle function [6]. Alterations of the maxilla, the mandible, as well as the tongue’s size, position, and geometry can lead to the obstruction of the retro-palatal region [29]. There is often excessive vertical facial growth—known as “adenoid facies” or “long face syndrome”—in children with OSA because of mouth breathing (Figure 1). Mouth breathing can be derived from various causes and is often linked to weak orofacial and whole-body muscle tone [15]. As seen in our case report, this patient’s mouth breathing was linked to an open mouth and reclined head posture with the downward and backward position of the mandible, establishing a Class II malocclusion (Figure 1) [30]. Reported effects on dental occlusion are shown in increased overjet, reduced overbite, unilateral crossbite (revealing a chin deviation extraorally), and open bite (Figure 1) [31]. The associated low tongue posture has a high impact on maxillary transversal growth development with skeletal manifestations (e.g., high, V-shaped maxilla) leading to pronounced rugae palatinae (Figure 1). Low tongue posture has a major impact on the growing structures compared to wrong tongue function (visceral swallowing with facial grimaces) since the total time of swallowing is too short to affect growing craniofacial structures [18]. Due to a short lingual frenulum (see ankyloglossia in Figure 1), the mobility of the tongue is restricted. This, in turn, could result in an insufficient upward tongue force to the maxilla and missing stimulation of the intermaxillary synchondrosis, which leads to the development of a high and narrow palatal arch. As a result, the airway space is reduced because of the increased narrowing of the nasal floor [19]. The mandible may also be affected, experiencing a forward pull that stimulates growth by restricted tongue mobility, resulting in a skeletal Class III malocclusion with a straight or prognathic profile (Figure 1) [32].

An enlargement of the tongue (macroglossia, Figure 1) is observed in many children diagnosed with OSA, particularly among those with Down syndrome. Assessing tongue size can be challenging, but indicative signs of significant enlargement include the posterior aspect of the tongue displacing the soft palate. Additionally, fatty infiltration of the tongue is frequently noted in obese children with OSA compared to age- and weight-matched controls without OSA [21].

Macroglossia mostly correlates with low tongue posture among Down syndrome patients, and it often leads to a skeletal Class III malocclusion with an untypical straight or concave profile (Figure 1). The Mallampati score, linked with obesity-related factors, enlarged tongue size, and an imbalanced tongue/mandible volume ratio, may be influenced by the impact of obesity on tongue dimensions and upper airway constriction. This score, outlined in Figure 1, serves as a predictive tool for pediatric OSA, predominantly reflecting pharyngeal obstruction [33].

The presented craniofacial and orofacial anomalies of the POSADEF aim to optimize patient health care through enhanced collaboration and a better understanding of these craniofacial anomalies’ impacts on pediatric OSA. This examination form should be a simple visual representation to assist dentists, orthodontists, pediatricians, and otolaryngologists in diagnosing and managing pediatric OSA. Our clinical case report confirms the validity of this approach in training pediatricians, otolaryngologists, and dental colleagues in craniofacial anomalies and abnormal function associated with pediatric OSA. POSADEF, published here for the first time as an initial guideline model, could be helpful in identifying airway obstructions caused by anatomical (soft tissue hypertrophy, skeletal anomalies, and obesity) and functional factors (dysfunction and muscle hypotonia). Accordingly, the treatment approach should be based on the diagnosed etiology. Therefore, in the future, a prospective clinical study will be conducted and may need to be adjusted to meet the clinical needs of other disciplines if necessary.

## 5. Conclusions

The multifactorial pathogenesis of obstructive sleep apnea in children requires interdisciplinary management during diagnosis and treatment. This newly developed Pediatric Obstructive Sleep Apnea Diagnostic Examination Form (POSADEF) aims to provide a simple visual representation to assist dentists, orthodontists, pediatricians, and otolaryngologists in diagnosing craniofacial and orofacial anomalies, such as abnormal function, in obstructive sleep apnea. A prospective clinical study is planned to evaluate the diagnostic examination form within the context of interdisciplinary treatment planning.

## Figures and Tables

**Table 1:** Risk factors (phenotypes) for persistent pediatric OSA after adenotonsillectomy.

Risk Factors (Phenotypes) for Persistent Pediatric OSA					
	Features	Association with OSA	Study	Year	Type of Study
**Craniofacial anomaly**	Adenoid facies (long face, mouth breathing, lip competence, orofacial muscle hypotonia)	Longer lower anterior face height [11,12] Long face [13] Mouth breathing [14,15] Muscle tone of the lips [16] Lower tongue strength [15]	Sutherland et al. Yap et al. Durdik et al. Bokov et al. Villa et al. Correa et al. Villa et al.	2020 2019 2018 2022 2017 2020 2017	Cohort study Case–control Cross-sectional study Case–control Randomized controlled trial Case–control Randomized controlled trial
	Mandibular retrognathia	Skeletal Class II pattern with a reduced mandibular length [17] Juvenile idiopathic arthritis (JIA)	Cozza et al. Di Francesco et al. Ma et al.	2004 2012 2022	Case–control Cohort study Cohort study
	Narrow palatal arch	Narrower maxillary arch [11] Palatal crossbite and reduced transverse maxillary width [18]	Yap et al. Katyal et al.	2019 2013	Case–control Case–control
	Class II occlusion	Increased frequency of Class II molar relationship [11]	Yap et al.	2019	Case–control
	Lateral crossbite	Increased frequency of lateral crossbite [11]	Yap et al.	2019	Case–control
**Orofacial anomaly**	Short lingual frenulum (ankyloglossia)	Reduced tongue mobility [19,20]	Yuen et al. Camañes-Gonzalvo et al.	2022 2024	Case–control Systematic review
	Macroglossia	Tongue size (macroglossia) [21] The imbalance between tongue and mandible volume [16]	Fleck et al. Correa et al.	2018 2020	Review Case–control study
**Muscular disorder/dysfunction**	Mouth breathing Reduced muscle tone	Reduced tongue tone may be a consequence of oral breathing and delayed chewing [15]	Villa et al.	2017	Randomized controlled trial
	Abnormal swallowing pattern	Tongue thrusting and abnormal swallowing patterns caused by a persistent mouth breathing posture [15]	Villa et al.	2017	Randomized controlled trial
**Obesity**	Body mass index	Reduction in BMI [22]	Andersen et al.	2019	Longitudinal study
	Tongue	Fatty infiltration of the tongue [21]	Fleck et al.	2018	Review

OSA = obstructive sleep apnea.

## Data Availability

The original contributions presented in the study are included in the article, further inquiries can be directed to the corresponding author.

## Abbreviations

OSA	Obstructive sleep apnea
SRBD	Sleep-related breathing disorder
SRBD-PSQ	Sleep-Related Breathing Disorder of the Pediatric Sleep Questionnaire
FR-2	Fränkel 2 appliance
MT	Myofunctional therapy
